# Thermographic Study of AZ31B Magnesium Alloy under Cyclic Loading: Temperature Evolution Analysis and Fatigue Limit Estimation

**DOI:** 10.3390/ma13225209

**Published:** 2020-11-18

**Authors:** Shaofei Guo, Xuesong Liu, Hongxia Zhang, Zhifeng Yan, Zhongdian Zhang, Hongyuan Fang

**Affiliations:** 1 State Key Laboratory of Advanced Welding and Joining, Harbin Institute of Technology, Harbin 150001, China; s.f.guo@hotmail.com (S.G.); zhangzhongdian@sina.com (Z.Z.); hyfang@hit.edu.cn (H.F.); 2 College of Materials Science and Engineering, Taiyuan University of Technology, Taiyuan 030024, China; hongxzhang@163.com (H.Z.); yanzhifeng@tyut.edu.cn (Z.Y.)

**Keywords:** thermography, temperature evolution, fatigue limit, magnesium alloy

## Abstract

In this paper, infrared thermography was employed to study the fatigue process of AZ31B magnesium alloy. In order to eliminate the interference caused by the temperature rise of the fixture, a data processing method was proposed, which is based on a special model to describe the temperature change of the specimen. Based on the temperature data after processing, the temperature evolution indicates that AZ31B magnesium alloy has undergone cyclic hardening during fatigue. Three different temperature indicators were selected to evaluate the fatigue limit based on the evolution curve after processing. In addition, the experimental results showed that the temperature data processed by the proposed method can be used to estimate the fatigue limit of AZ31B magnesium alloy. Experiments were performed for both extrusion and transverse directions in consideration of the anisotropy of the AZ31B.

## 1. Introduction

Infrared thermography offers a novel perspective for fatigue research [1] and has great potential in fatigue performance testing [2]. Different from the traditional statistical methods, thermography is based on the temperature rise of the material caused by cyclic loading, whose mechanism consists of the close relationship between the energy dissipation and the microstructural evolution inside materials during fatigue [3,4,5].

Many fatigue studies using infrared thermographic methods have been carried out, including estimating fatigue limit [6,7,8], predicting fatigue life [9,10,11], monitoring fatigue damage evolution [1,12,13,14], studying the effect of free surface and mean stress [15], characterizing microplasticity [16,17,18], and modeling fatigue crack growth [19,20,21], etc.

Infrared thermography has the ability to obtain fatigue limit by consuming only one specimen in less than one day [6,7]. It makes the thermographic methods attractive compared to the traditional statistical methods.

As two classic fatigue limit evaluation methods of infrared thermography, the Luong method [6] and the Risitano method [7] must be mentioned here. Luong [6] observed that the temperature increment of materials at loads above the fatigue limit is significantly higher than that below the fatigue limit. On the basis of explaining the mechanism of this phenomenon, Luong proposed to fit the data points in the temperature–stress plotting using two straight lines, one corresponding to the portion above the fatigue limit and the other one corresponding to the portion below the fatigue limit. The stress value corresponding to the intersection of the two fitted lines is identified as the fatigue limit.

Risitano et al. [7] hold a different perspective on how to select thermal data for fatigue limit assessment. They believe that the heat production of materials below the fatigue limit is too weak to be considered. Thus, in the method proposed by Risitano et al., only one straight line is left to fit the temperature data above the fatigue limit, and the fatigue limit corresponds to the intersection of the fitted line and the coordinate axis representing the applied load.

In 2015, De Finis et al. [8] proposed a thermographic methodology with a unique strategy to rapidly estimate fatigue limits. As preparation, a special linear filter program is used to eliminate the external interference on the self-heating measurement. Subsequently, a robust analysis was used to determine the fatigue limit. Its idea is that self-heating above the fatigue limit will be statistically higher than self-heating below the fatigue limit.

Meneghetti et al. [22,23,24,25] developed an energy method to evaluate the fatigue limit of AISI 304L stainless steel. A new fatigue damage index was proposed in the method of Meneghetti. According to the local energy balance theory, this index is equal to the energy dissipation in unit volume of material per unit time, which is independent of thermal and mechanical boundary conditions. Moreover, the results of many tests (about 100 sets of data) can be normalized (synthesized into a unique scatter band). These tests are conducted under different conditions including different testing conditions (stress-controlled or strain-controlled) and different geometry of specimens (plain or notched).

In order to apply a stable cyclic load, the specimen and the fixture are in close contact during the fatigue test, and the heat exchange between the two is unavoidable [26,27]. This makes the temperature of the specimen inevitably disturbed by the temperature change of the fixture during the test [28]. In theory, the fatigue damage of a material is only related to the temperature rise of the material itself caused by energy dissipation [1]. The interference from the fixture is likely to cause the result of temperature-based fatigue performance evaluation to be ambiguous [8]. How to overcome the interference of the fixture has always been a troublesome problem.

In this paper, infrared thermography was employed to study the fatigue process of AZ31B magnesium alloy. At first, a data processing method was adopted to eliminate interference from the fixture, which is based on a special model to describe the temperature change of the specimen. After that, the temperature evolution of the AZ31B magnesium alloy during fatigue was analyzed based on the processed temperature data. Finally, the fatigue limit of AZ31B was evaluated on the basis of temperature evolution analysis. Experiments were performed for both extrusion and transverse directions in consideration of the anisotropy of the AZ31B.

## 2. Experimental Material and Setup

The experimental material used in this study was a commercial extruded AZ31B magnesium alloy sheet. The chemical composition is shown in Table 1, in which Bal. (balance) means the predominating element. The basic mechanical properties of AZ31B were determined beforehand by uniaxial tensile tests. The tensile strength, yield strength and elongation in the extrusion direction are 251 MPa, 145 MPa and 9%, respectively. In addition, the tensile strength, yield strength and elongation in the transverse direction are 232 MPa, 130 MPa and 12%, respectively. Meanwhile, some thermophysical properties of AZ31B are given. The density *ρ* is 1770 kg/m^3^, the specific heat capacity *C* is 1000 J/(kg·K), and thermal conductivity *k* is 96 W/(m·K).

**Figure 1:**
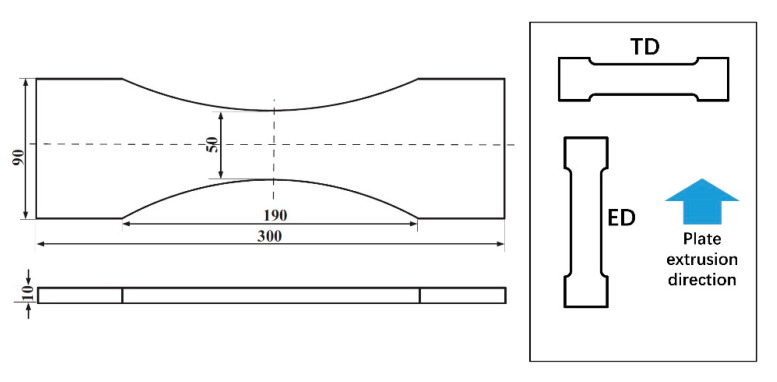
Specimen geometry (mm) and sampling direction.

Geometry of the specimen was designed according to the Chinese national standard GB/T-3075-2008 [29], as shown in Figure 1. Thickness of the specimen is 10 mm. Considering that the extruded AZ31B magnesium alloy owns obvious anisotropy, two types of specimens were prepared in this study, which are denoted as ED (extrusion direction) and TD (transverse direction). A schematic illustration of specimen orientation is given in Figure 1. Spark-cutting was used to machine the specimens from the plate. After that, the specimens were polished with 800, 1000 and 1500 grit metallographic sandpaper in order to get a smooth surface. Black matte paint was evenly sprayed on the surface of the fatigue specimen with the purpose of increasing the thermal emission rate.

**Figure 2:**
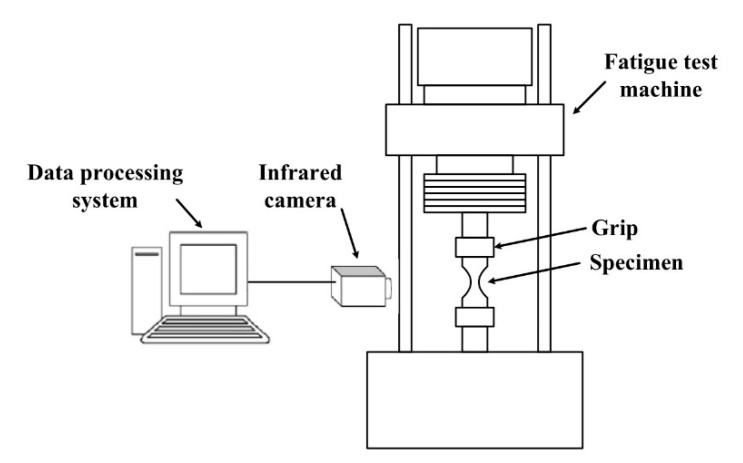
Schematic diagram of the experimental setup.

A schematic diagram of the experimental setup is shown in Figure 2. A PLG-200D high-frequency electromagnetic resonance fatigue tester (Changchun New Testing Machine Co., Ltd., Changchun, China) was employed in this study. The stress control mode was adopted. The load waveform was a sine wave. Additionally, the load frequency was approximately 100 Hz. The stress ratio was chosen to be *R* = 0.1 ($\sigma_{\min}/\sigma_{\max}$, where *σ*_min_ is the minimum stress and *σ*_max_ is the maximum stress). Load levels were classified according to the maximum value of the nominal stress at the center of the specimen with the smallest cross section, *σ*_max_. For the extrusion direction, the load level ranges from 70 MPa to 150 MPa. For the transverse direction, the load level ranges from 70 MPa to 140 MPa.

An infrared camera (InfraTec VarioCAM hr, InfraTec, Dresden, Saxony, Germany) was employed to record in real-time the surface temperature along with the ambient temperature during the fatigue test. The sampling speed of the camera was 50 frames per second. The temperature sensitivity is less than 0.08 K at 300 K. A software (IRBIS^®^3, InfraTec, Dresden, Saxony, Germany) provided by the equipment supplier was used together with the infrared camera to record the temperature changes on the surface of the specimen as a sequence of thermal images. The thermal image is stored in chronological order in the form of a digital matrix. The size of the digital matrix is 384 × 288, and the value of each element is the temperature at the corresponding position. Thanks to the infrared camera, we can thoroughly examine the entire surface of the specimen at each point (pixel by pixel) and the temperature variation over time. The spatial resolution is from 0.74 mm to 0.78 mm square pixels which is affected by the scanner distance. The infrared camera is regularly calibrated by the manufacturer to ensure its accuracy.

## 3. Temperature Data Processing to Eliminate Interference from the Fixture

The temperature of the fixture usually changes with time during the fatigue test [27]. This will affect the temperature of the specimen and may lead to the result of the temperature-based fatigue performance evaluation to be ambiguous [8]. Some researchers had installed a circulating cooling device around the fixture of the fatigue tester to suppress the temperature rise at both ends of the specimen [28]. Different from this kind of experimental approach, a data processing method is proposed and used to control the temperature rise of the fixture in this work.

### 3.1. Thermal Model for Middle Portion of the Specimen

**Figure 3:**
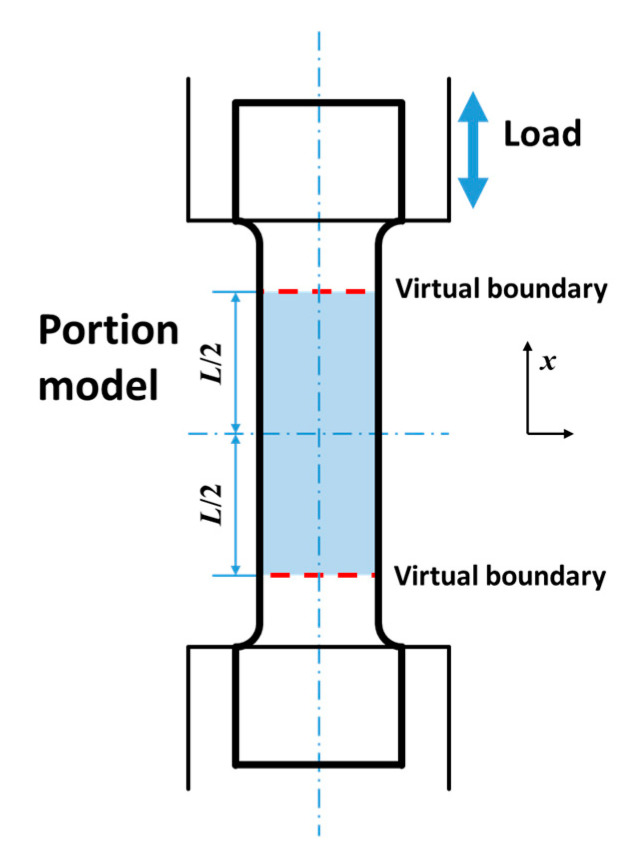
Schematic of the portion of the specimen for build the thermal model.

A thermal model needs to be established to describe the heat conduction process of the specimen during the fatigue test, which is the basis of subsequent analysis [30]. Different from the temperature model for the entire specimen used in many documents [4,15], the middle portion (the area marked with light blue) is used as the object to build the thermal model in this work, as schematically illustrated in Figure 3. Two symmetrical virtual boundary lines (the red dashed lines) are set artificially on both sides of the specimen center. The setting of two boundary lines can be regarded as a re-division of the entire fatigue specimen. In addition, the middle portion of the specimen can be regarded as a smaller specimen that is bordered by two dashed lines. In contrast, the part of the specimen that is outside the boundary can be regarded as an extended fixture. In this way, as long as all fatigue specimens are divided in the same way, it can be guaranteed that all tests use portion specimens with the same size.

In order to describe a given heat conduction process, a complete mathematical model should include heat diffusion equations, initial conditions and boundary conditions [31]. Among them, the role of the heat diffusion equation is to describe the law of temperature change of objects.

From the perspective of non-equilibrium thermodynamics, fatigue damage can be regarded as a quasi-static process, in which the thermodynamic state of the material undergoes irreversible evolution under continuous cyclic loading. This irreversible evolution can be generally represented by a finite set of variables, which include the absolute temperature *T*, the small strain tensor *ε*, and a vector *α* including *N* internal variables [32,33]. Combining the local forms of the first and second principles of thermodynamics, and assuming the Fourier heat conduction law, the thermal diffusion equation describing local temperature changes can be written as follows:

(1) $$\rho C\dot{T}-\mathrm{div}\left(k\mathrm{grad}\left(T\right)\right)=d_{1}+s_{the}+s_{thc}+r_{ext}$$

where *ρ* is the mass density, *C* is the specific heat, and *k* is the isotropic heat conduction tensor. The left-hand side of this equation is a differential operator applied to *T*, and the right-hand side is a collection of different heat sources, which are in turn: the intrinsic dissipation *d*_1_, the thermoelastic source *s_the_*, the other possible thermomechanical coupling sources *s_thc_* and the external volume heat supply *r_ext_*.

For general fatigue tests at room temperature, some hypotheses can be made to simplify the local thermal diffusion equation [32,34].

- During the entire fatigue test, the parameters *ρ*, *C*, and *k* are material constants, which are independent of time and other state variables.
- Ignore other thermocouple sources other than thermoelastic sources, *s_thc_* = 0, as it can be considered that the low temperature rise of the specimen during the fatigue test will not cause the change of the material microstructure.
- The convection terms of the total time derivative of the temperature were neglected.
- The external volume heat supply does not change over time. The equilibrium temperature field *T*_0_ verifies $-k\Delta T_{0}=r_{ext}$.

Based on the above assumptions, the heat diffusion equation describing local temperature changes can be simplified into the following form by writing $\theta =T-T_{0}$:

(2) $$\rho C\frac{\partial \theta }{\partial t}-k\Delta \theta =d_{1}+s_{the}$$

where *θ* symbolizes the temperature variation.

In this study, we take the average temperature change within 1 s (about 100 load cycles). Under this consideration, the temperature fluctuations caused by thermoelastic effects cancel each other out, and the intrinsic dissipation source is the only internal heat source to be considered [32].

(3) $$\rho C\frac{\partial \theta }{\partial t}-k\Delta \theta =d_{1}$$

Since the temperature gradient along the length of the fatigue specimen is much larger than the other two dimensions (thickness and width), it is reasonable to adopt a one-dimensional thermal diffusion equation to describe the temperature evolution of the specimen during the fatigue test [4,35]. Set the length direction to the *X*-axis. The one-dimensional thermal diffusion equation can be obtained by integrating the temperature of each cross section of the specimen [36,37]:

(4) $$\rho C\frac{\partial \overset{=}{\theta }}{\partial t}-k\left(\frac{\partial^{2}\overset{=}{\theta }}{\partial x^{2}}+\frac{1}{S}\frac{dS\partial \overset{=}{\theta }}{dx\partial x}\right)={\overset{=}{d}}_{1}$$

where S(x)=e·w(x) represents the cross-section area of the specimen, *e* represents the thickness of the specimen, and w(x) represents the width of the specimen; $\frac{1}{S}\frac{dS\partial \overset{=}{\theta }}{dx\partial x}$ represents the influence of the specimen shape on the temperature distribution; $\overset{=}{\theta }\left(x,t\right)$ represents the average temperature variation over the thickness–width obtained from the thermal image.

The total heat exchange between the specimen and the outside generally includes heat exchange through the two ends and heat exchange through the lateral [8]. The heat exchange through the two ends of the specimen is dominated by heat conduction between the specimen and the fixture. This close contact between metals has very low heat transfer resistance, which means a strong heat exchange capacity. In contrast, the lateral of the specimen exchanges heat with the surrounding environment through thermal convection and radiation. At the same temperature difference, the amount of heat lost in a unit of time in this way is relatively limited. For simplicity, the heat exchange on the lateral of the specimen was ignored. In the work of Yang et al. [38], the same assumption was made to simplify the heat equation.

Equation (4) is a heat conduction differential equation, which describes the general law of the temperature change of the specimen during the fatigue process. However, Equation (4) alone does not address the specific characteristics of temperature evolution in a particular test. In order to identify a certain heat conduction process of interest, Equation (4) needs to be further defined [31].

The initial conditions explain the temperature distribution at the beginning of the heat conduction process. The heat transfer process of interest in this study starts at the moment the fatigue test begins. At this moment, heat starts to be generated inside the material and causes the temperature of the specimen to change. Therefore, the initial conditions of the model are set according to the temperature distribution on the surface of the specimen at the starting moment of cyclic loading, $\overset{=}{\theta }\left(x,t=0\right)={\overset{=}{\theta }}_{exp}\left(x,t=0\right)$.

The boundary conditions explain the characteristics of the thermal process on the boundary of the object. Here, the temperature change over time on two virtual boundaries is selected as the boundary condition of the model, $\overset{=}{\theta }\left(x=\pm \frac{L}{2},t\right)={\overset{=}{\theta }}_{exp}\left(x=\pm \frac{L}{2},t\right)$, which is to set the thermal boundary conditions according to the first type [31].

Therefore, the temperature evolution of the part between the two boundaries during the entire fatigue test can be modeled as follows:

(5) $$\{\begin{array}{c} \rho C\frac{\partial \overset{=}{\theta }}{\partial t}-k\left(\frac{\partial^{2}\overset{=}{\theta }}{\partial x^{2}}+\frac{1}{S}\frac{dS\partial \overset{=}{\theta }}{dx\partial x}\right)={\overset{=}{d}}_{1}\\ \overset{=}{\theta }\left(x,t=0\right)={\overset{=}{\theta }}_{exp}\left(x,t=0\right)\\ \overset{=}{\theta }\left(x=\pm \frac{L}{2},t\right)={\overset{=}{\theta }}_{exp}\left(x=\pm \frac{L}{2},t\right) \end{array}$$

where *s* is the heat source term of the model, which is equal to the mean of energy dissipation ${\overset{=}{d}}_{1}$; ${\overset{=}{\theta }}_{exp}$ is the actual temperature determined from the thermal image; *L* is the distance between the two boundaries.

### 3.2. Separation of Raw Temperature Data

As the model representing the temperature change of the specimen during the fatigue test, Equation (5) can be separated into two parts that can be linearly superimposed according to the mathematical superposition principle [37].

(6) $$\overset{=}{\theta }\left(x,t\right)={\overset{=}{\theta }}_{d}\left(x,t\right)+{\overset{=}{\theta }}_{b}\left(x,t\right)$$

where

(7) $$\{\begin{array}{c} \rho C\frac{\partial {\overset{=}{\theta }}_{d}}{\partial t}-k\left(\frac{\partial^{2}{\overset{=}{\theta }}_{d}}{\partial x^{2}}+\frac{1}{S}\frac{dS\partial {\overset{=}{\theta }}_{d}}{dx\partial x}\right)={\overset{=}{d}}_{1}\\ {\overset{=}{\theta }}_{d}\left(x,t=0\right)=0\\ {\overset{=}{\theta }}_{d}\left(x=\pm \frac{L}{2},t\right)=0 \end{array}$$

(8) $$\{\begin{array}{c} \rho C\frac{\partial {\overset{=}{\theta }}_{b}}{\partial t}-k\left(\frac{\partial^{2}{\overset{=}{\theta }}_{b}}{\partial x^{2}}+\frac{1}{S}\frac{dS\partial {\overset{=}{\theta }}_{b}}{dx\partial x}\right)=0\\ {\overset{=}{\theta }}_{b}\left(x,t=0\right)={\overset{=}{\theta }}_{exp}\left(x,t=0\right)\\ {\overset{=}{\theta }}_{b}\left(x=\pm \frac{L}{2},t\right)={\overset{=}{\theta }}_{exp}\left(x=\pm \frac{L}{2},t\right) \end{array}$$

Equation (7) is a non-homogeneous equation with a homogeneous boundary condition, where the source term ${\overset{=}{d}}_{1}$ is equal to the source term in Equation (5). Homogeneous boundary conditions of ${\overset{=}{\theta }}_{d}\left(x,t\right)$ mean that the temperature rise on both boundaries is always zero. At the same time, for the non-homogeneous equation, the heat source term ${\overset{=}{d}}_{1}$ in Equation (7) is equal to the heat source term in Equation (5). Combining the two aspects, the physical meaning of ${\overset{=}{\theta }}_{d}\left(x,t\right)$ can be summarized as the temperature rise of the specimen obtained by performing the test under an ideal experimental condition. That is, when the cyclic load causes the heating inside the material, the temperature rise on the boundary of the specimen is always kept at zero at the same time ${\overset{=}{\theta }}_{d}\left(x=\pm \frac{L}{2},t\right)=0$. On the other hand, the heat source term always remains zero in Equation (8). The initial conditions and the upper and lower boundary conditions are the same as $\overset{=}{\theta }\left(x,t\right)$, which are equal to the actual measurement results. So then, ${\overset{=}{\theta }}_{b}\left(x,t\right)$ represents the temperature change caused by the boundary conditions, that is, the interference of the temperature rise of the fixture during the fatigue test.

### 3.3. Algorithm to Eliminate Interference from Fixture

Fatigue damage is only related to the energy dissipation of the material itself. Here, the author proposes to evaluate the fatigue performance based on ${\overset{=}{\theta }}_{d}\left(x,t\right)$ in order to eliminate the interference of the temperature rise of the fixture.

${\overset{=}{\theta }}_{d}\left(x,t\right)$ can be calculated by ${\overset{=}{\theta }}_{d}\left(x,t\right)=\overset{=}{\theta }\left(x,t\right)-{\overset{=}{\theta }}_{b}\left(x,t\right)$. On the one hand, $\overset{=}{\theta }\left(x,t\right)$ is known, which can be obtained by modeling the thermographic data according to Equation (5). On the other hand, ${\overset{=}{\theta }}_{b}\left(x,t\right)$ can be calculated by the finite difference method. The heat source term of Equation (8) is zero. Additionally, its initial and boundary conditions are the same as the initial and boundary conditions of $\overset{=}{\theta }\left(x,t\right)$, respectively, which can be extracted from the known temperature model.

A procedure of excluding the temperature rise caused by the boundary conditions from the raw temperature data is given as follows:

1. According to the temperature data obtained by the infrared thermal camera, a one-dimensional temperature model is established to describe the temperature change of the specimen during the fatigue process, which is Equation (5).
2. Extract the initial condition $\overset{=}{\theta }\left(x,t=0\right)={\overset{=}{\theta }}_{exp}\left(x,t=0\right)$ and boundary condition $\overset{=}{\theta }\left(x=\pm \frac{L}{2},t\right)={\overset{=}{\theta }}_{exp}\left(x=\pm \frac{L}{2},t\right)$ from the obtained temperature model $\overset{=}{\theta }\left(x,t\right)$.
3. According to Equation (8), substitute the initial and boundary conditions extracted in the previous step, and set the heat source term to zero. Then, ${\overset{=}{\theta }}_{b}\left(x,t\right)$ is obtained by numerical calculation.
4. ${\overset{=}{\theta }}_{d}\left(x,t\right)$ is obtained by ${\overset{=}{\theta }}_{d}\left(x,t\right)=\overset{=}{\theta }\left(x,t\right)-{\overset{=}{\theta }}_{b}\left(x,t\right)$.

## 4. Results and Discussion

### 4.1. Results of Traditional Fatigue Test

**Figure 4:**
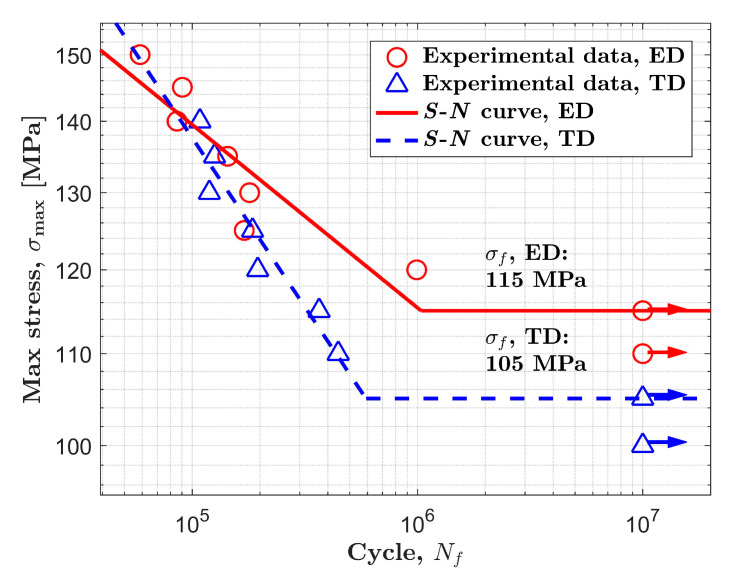
*S*–*N* curves of AZ31B magnesium alloy, ED and TD.

Figure 4 is the *S*–*N* curves of the AZ31B magnesium alloy, in which *σ*_max_ represents the maximum stress and *N_f_* represents the number of cycles of the load. Obviously, bilinearity can be observed from the *S*–*N* curve in both ED and TD directions. It seems that the AZ31B magnesium alloy possesses a definite fatigue limit, although it is generally believed that there is no fatigue limit for non-ferrous alloys. Similar conclusions have been reported by other researchers [39,40]. In this study, the fatigue strength at 10^7^ cycles was defined as the fatigue limit. According to Figure 4, the fatigue limit of the AZ31B magnesium alloy in the extrusion direction is 115 MPa, and the material in the transverse direction has a lower fatigue limit of 105 MPa.

### 4.2. Temperature Evolution Analysis

**Figure 5:**
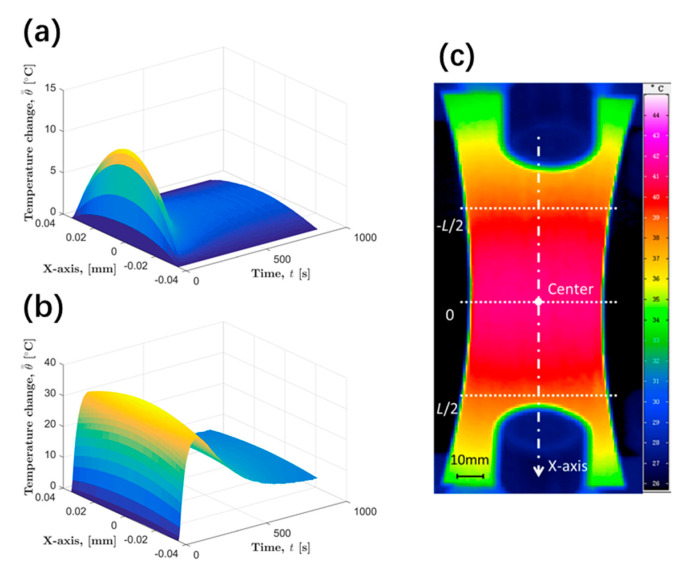
Example of specimen temperature change that has been modeled, (**a**) after processing, (**b**) before processing, (**c**) two set virtual boundaries.

Figure 5a exhibits an example of the temperature evolution of specimen that the boundary temperature has been controlled using the proposed algorithm. In addition, the corresponding temperature evolution before data processing is given in Figure 5b. This example was carried out with the specimen in the extrusion direction under a load of 145 MPa. $\overset{=}{\theta }=T-T_{0}$ represents the temperature increment. The one-dimensional portion temperature model introduced in Section 3.1 has been built by taking the average temperature of each cross section of the specimen. Two virtual boundaries are set on the specimen, as shown in Figure 5c. The distance *L* between the two boundaries of some models is set to 70 mm in order to cover as much temperature information as possible on the surface of the specimen. As can be seen in the figure, the temperature increment of the specimen on the two boundaries always remains at zero during the entire test after processing. This is consistent with the theoretically expected result. In contrast, the temperature data before processing have a changing boundary temperature.

**Figure 6:**
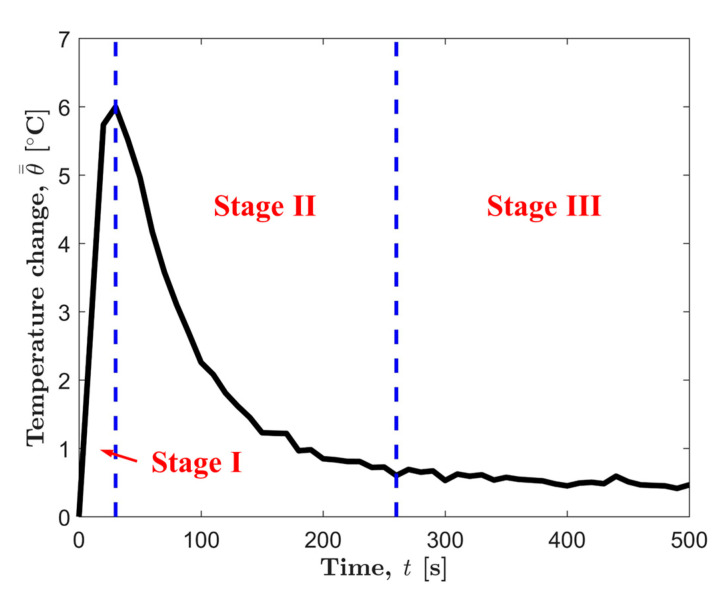
Typical temperature evolution curve processed by the superposition filter.

Figure 6 shows a typical center temperature evolution curve of AZ31B magnesium alloy before the macro fatigue cracks appear. It can be found that the center temperature evolution after processing can be divided into three stages: the temperature rise stage (Stage I), the temperature drop stage (Stage II), and the stable stage (Stage III).

**Figure 7:**
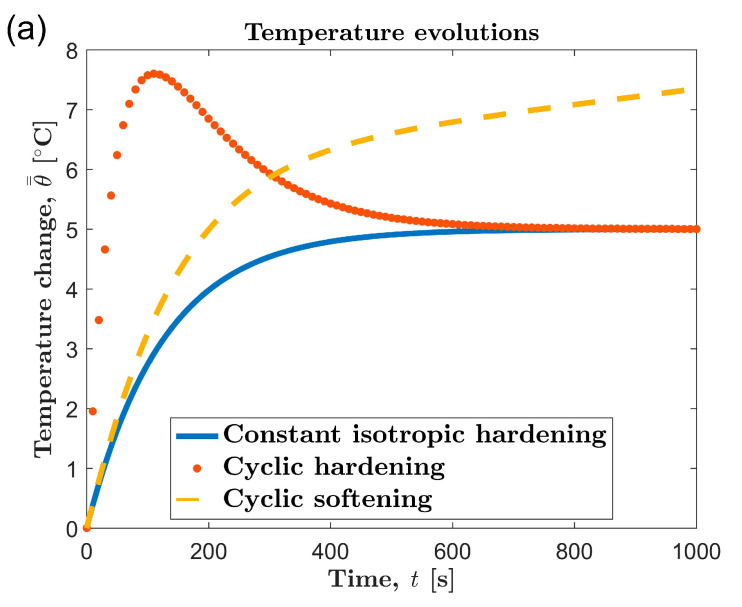

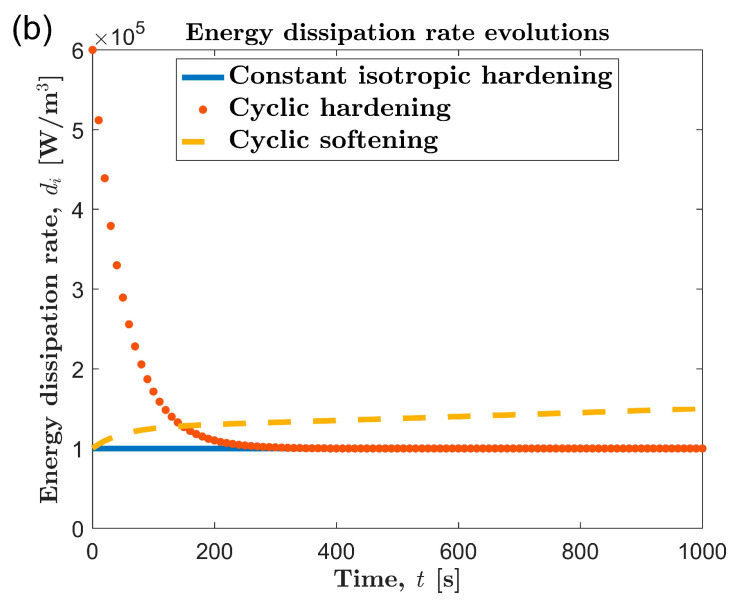
(**a**) Temperature evolution curves and (**b**) energy dissipation rate evolution curves for different hardening types.

In 2009, Doudard et al. [41] found that the trend of the temperature evolution curve during fatigue testing is related to the cyclic hardening/softening mode of the material. According to the different cyclic hardening/softening modes, the temperature evolution curve during the fatigue process can be classified into three representative trends, as shown in Figure 7a. In addition, the different types of temperature evolution curves result from the difference in the evolution of heat production per unit time, as shown in Figure 7b. Obviously, the three-stage temperature evolution of AZ31B magnesium alloy is consistent with the temperature evolution curve of cyclic hardening materials.

The heat generation per unit time under cyclic loading is related to plastic deformation. Generally, it can be considered that the greater the plastic deformation, the greater the thermal energy converted from mechanical work per unit time. The AZ31B magnesium alloy is a cyclic hardening material [42], which has a large plastic deformation in the initial stage of cyclic loading. With the cyclic loading, the plastic deformation becomes smaller and smaller and gradually becomes saturated. Correspondingly, as shown in Figure 7b, the heat generation of AZ31B during the fatigue test also showed a gradual decrease and saturation trend.

From the perspective of dynamic energy balance, temperature is the result of competition between the internal heat generation of the specimen and the heat exchange between the specimen and the outside [33]. Before the test starts, after sufficient cooling, the temperature of the specimen and the surrounding environment remains the same. When the fatigue test begins, mechanical energy is converted into thermal energy under the effect of cyclic load, and the heat generation inside the specimen begins to increase. The thermal equilibrium before the test is broken, and, as a result, the temperature of the specimen starts to rise. The temperature curve after data processing is equivalent to the result obtained in a constant ideal environment. An increase in the temperature of the specimen means that the temperature of the specimen is higher than the ambient temperature, so the specimen also begins to lose heat due to the temperature difference. Throughout the first stage, the rate of heat production per unit time is greater than the rate of heat loss, resulting in a continuous rise in time and temperature.

As the temperature of the specimen increases, the temperature difference between the specimen and the environment becomes larger and larger, and the heat loss per unit time becomes more. On the other hand, due to cyclic hardening, the plastic deformation capacity of AZ31B continues to decrease, and the heat generated per unit time also gradually decreases. Until the heat dissipated to the outside matches the heat generated inside, the temperature of the specimen no longer rises, which corresponds to the boundary between the first and second stages. Subsequently, under the effect of work hardening, the heat generation rate continued to decline. The gradual decline in heat generation rate cannot offset heat loss per unit time. The specimen temperature began to decrease. This corresponds to the temperature drop phase, phase II of the AZ31B temperature evolution curve.

As a result of the temperature drop, the temperature difference between the specimen and the surrounding environment gradually decreases, and the amount of heat lost in the unit time becomes smaller and smaller. With the progress of work hardening, the plastic deformation of AZ31B tends to a constant saturation value. The heat loss of the specimen to the outside and the internal heat generation begin to enter a new dynamic equilibrium. The temperature of the specimen no longer changes with the temperature, corresponding to the third stage of the temperature evolution curve.

**Figure 8:**
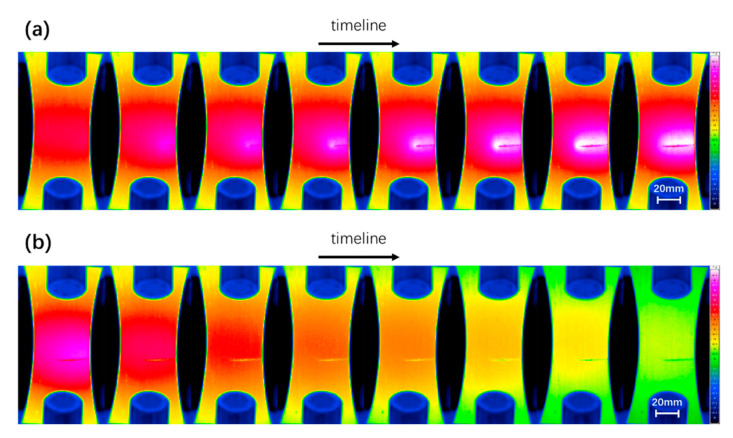
A series of thermal images of (**a**) the macro crack propagation and (**b**) the natural cooling after final failure.

The constant temperature (Stage III) will continue until the fatigue cracks become visible on the thermal image. Thereafter, the specimen will experience a period of rapid temperature rise caused by crack propagation along with a period of natural cooling after final failure, as shown in Figure 8. In some studies, these two phases serve as the last two phases of temperature evolution [42]. This work will not consider these two parts. The reason is that the appearance of macro cracks damages the integrity of the fatigue specimen to a certain extent. However, the one-dimensional thermal diffusion equation used in this paper for modeling is for a complete specimen. In fact, the temperature evolution curves of the first three stages of AZ31B have provided enough experimental data to be used in the study of material fatigue properties.

### 4.3. Fatigue Limit Estimation Based on Processed Temperature Data

The theoretical basis of the temperature-based fatigue limit assessment lies in the transformation of the energy dissipation mechanism caused by the transformation of the fatigue damage mechanism, which manifests as an inflection point in the stress–temperature relationship curve near the fatigue limit [6,33]. For the fatigue life, the transformation of the fatigue damage mechanism is manifested as the bilinearity of the *S*–*N* curve, which can be understood to be that an inflection point exists in the *S*–*N* curve near the fatigue limit. It is generally believed that there is no fatigue limit for non-ferrous alloys. However, it is the opinion of authors that as long as the *S*–*N* curve of material exhibits bilinearity, it is possible to try to evaluate its fatigue limit with a temperature-based method. As the bilinearity of the *S*–*N* curve means that there is a shift in the fatigue damage mechanism, the transformation of fatigue damage mechanism will be reflected in the transformation of energy dissipation mechanism, and finally reflected in the stress–temperature relationship curve.

As shown in Figure 4, the *S–N* curve of the AZ31B magnesium alloy exhibits obvious bilinearity in both ED and TD directions. Therefore, it is reasonable to think that the temperature-based method is suitable for evaluating the fatigue limit of AZ31B magnesium alloy. In this section, the fatigue limit of AZ31B will be evaluated based on temperature data after eliminating the effects of the fixture.

**Figure 9:**
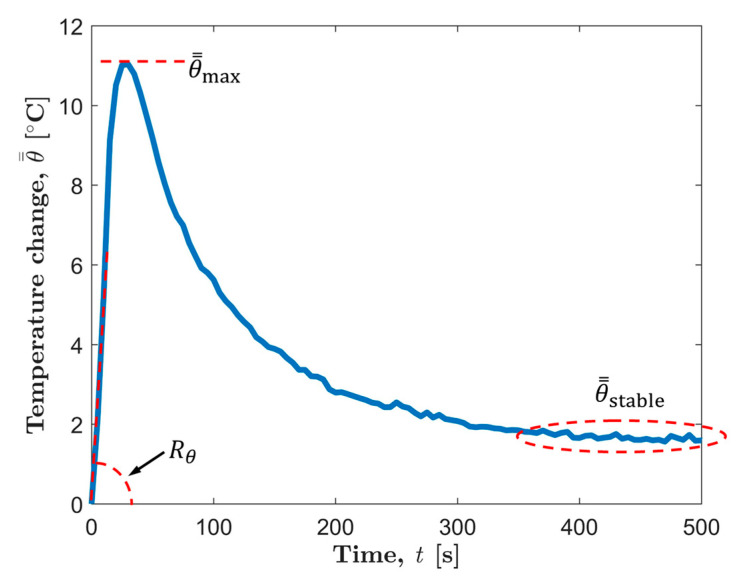
Thermal indicators for fatigue limit evaluation.

With reference to the published literature [26,43], three commonly used thermal indicators were selected for subsequent fatigue limit evaluation: the initial temperature rise slope, maximum temperature rise, and equilibrium temperature, as shown in Figure 9. The three selected thermal indicators are determined according to the central temperature evolution curve after processing. A dedicated Matlab program is used to automatically calculate these three thermal indicators according to the temperature evolution curve for all load levels. In particular, the initial temperature rise slope is calculated based on the temperature data of the first 10 s and is calculated using a linear fit [44]. The maximum temperature rise takes the maximum value of the temperature rise curve. The equilibrium temperature rise is calculated by averaging temperature data at approximately 100 s intervals during the temperature stable stage.

**Figure 10:**
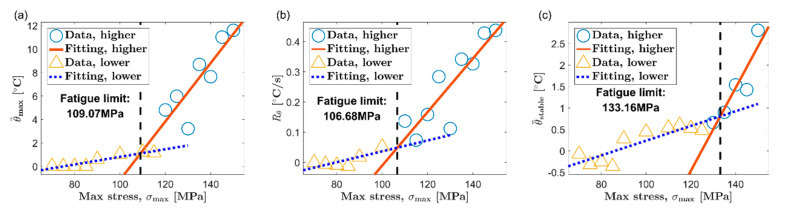
Fatigue limit evaluation using the Luong method, ED: (**a**) maximum temperature rise, ${\overset{=}{\theta }}_{\max}$; (**b**) initial temperature rise slope, $R_{\theta }$; (**c**) mean temperature rise of stable phase, ${\overset{=}{\theta }}_{\mathrm{stable}}$.

**Figure 11:**
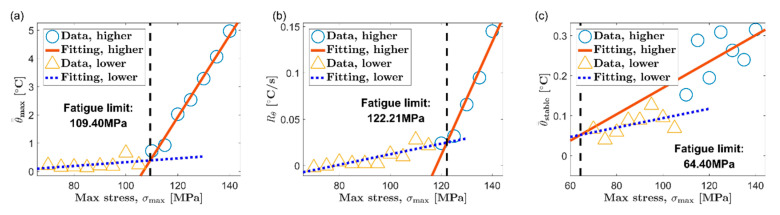
Fatigue limit evaluation using the Luong method, TD: (**a**) maximum temperature rise, ${\overset{=}{\theta }}_{\max}$; (**b**) initial temperature rise slope, $R_{\theta }$; (**c**) mean temperature rise of stable phase, ${\overset{=}{\theta }}_{\mathrm{stable}}$.

**Figure 12:**
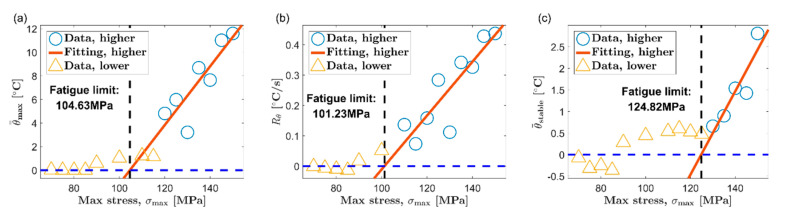
Fatigue limit evaluation using the Risitano method, ED: (**a**) maximum temperature rise, ${\overset{=}{\theta }}_{\max}$; (**b**) initial temperature rise slope, $R_{\theta }$; (**c**) mean temperature rise of stable phase, ${\overset{=}{\theta }}_{\mathrm{stable}}$.

**Figure 13:**
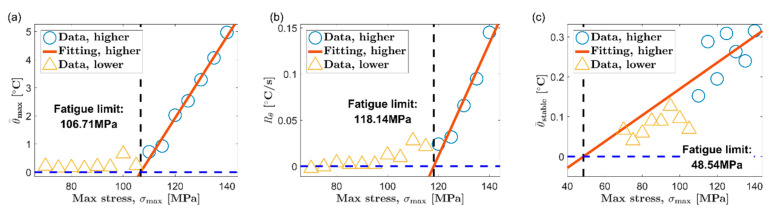
Fatigue limit evaluation using the Risitano method, TD: (**a**) maximum temperature rise, ${\overset{=}{\theta }}_{\max}$; (**b**) initial temperature rise slope, $R_{\theta }$; (**c**) mean temperature rise of stable phase, ${\overset{=}{\theta }}_{\mathrm{stable}}$.

Based on the thermal indicators obtained under different loads, two classic temperature-based fatigue limit assessment methods, Luong method [6] and Risitano method [7], are used to determine the fatigue limit of AZ31B magnesium alloy. Figure 10 and Figure 11 show the results of the Luong method for the extrusion direction (ED) and the transverse direction (TD), respectively. Additionally, the results of fatigue limit identified by the Risitano method are given in Figure 12 (ED) and Figure 13 (TD). The inflection point of the temperature indicator–stress curve is taken as a basis to distinguish whether the load at each data point is higher than the fatigue limit. The data points above the fatigue limit are represented by blue circles. The data points below the fatigue limit are represented by yellow triangles. In Figure 10 and Figure 11, for the results of the Luong method, the red straight line and the blue dotted line are linear fits to the data points above and below the fatigue limit, respectively. In Figure 12 and Figure 13, for the results of the Risitano method, the red straight line is the result of a linear fit to the data points above the fatigue limit, and the *X*-axis is highlighted by a blue horizontal dashed line.

The results of the Luong method and the Risitano method for fatigue limit estimation are summarized in Table 2. The error of the results of the temperature-based method relative to the traditional method is given at the same time. It can be seen that all the errors of the results using the maximum temperature rise ${\overset{=}{\theta }}_{\max}$ as the thermal indicator are all within ±10%, which is a satisfactory estimate. On the other hand, the error of the result based on the initial temperature rise slope $R_{\theta }$, relative to the traditional method, is about ±15%. Although this accuracy is not as good as the result of the maximum temperature rise, these results are basically acceptable.

A short experimental period is a significant advantage of the temperature-based method. The temperature evolution curve of AZ31B magnesium alloy will reach the maximum soon after the initial temperature rise stage. The cyclic loading can be stopped when the maximum temperature rise is reached, which will save experiment time to a great extent. In this paper, the initial temperature rise slope is calculated by linear fitting the temperature rise curve within 10 s after the fatigue loading starts, which refers to the published report [44]. This part of the temperature rise curve only accounts for a small part of the first temperature rise stage. Indeed, choosing the initial temperature rise slope can save more time compared to the maximum temperature rise. However, if different durations are selected, the initial temperature rise slope will be significantly different. This will further affect the evaluation results of the fatigue limit. In contrast, the maximum temperature rise can be determined according to the inflection point of the temperature evolution curve. Its definition is not disputed. Moreover, the maximum temperature rise is high enough to ignore the influence of random noise. Therefore, the authors think that the maximum temperature rise is a more promising thermal indicator for the fatigue limit assessment of AZ31B magnesium alloy based on temperature.

The temperature in the stable stage is a common thermal indicator and has been used in many studies [6,7,8]. Nevertheless, the stable temperature does not seem to be a very ideal choice for AZ31B magnesium alloy. As reported in many studies, many steels will directly enter the temperature stabilization phase after the first temperature rise phase. In contrast, after the initial temperature rise stage, the AZ31B magnesium alloy has to undergo a temperature drop stage to reach temperature equilibrium. More time is required to obtain the stable temperature for AZ31B. In addition, from the experimental results, the stable temperature of AZ31B increases slowly along with the load, which results in a smaller slope of the fitted line above the fatigue limit. This sometimes will lead to a poor fatigue limit evaluation, such as the results in Figure 11c and Figure 13c.

### 4.4. Discussion about the Portion Model

It should be emphasized that the fatigue limit evaluation results of the thermographic methods in Table 2 are all based on temperature data that the boundary temperature has been controlled using the proposed algorithm. In addition, this data processing method is based on the novel portion model that describes the temperature change of the specimen.

A considerable amount of heat in the specimen will flow to the fixture through contact heat transfer, which has a great influence on the temperature of the specimen during the fatigue test. If the model is established based on the entire specimen, the contact heat transfer coefficient between the specimen and the fixture must be quantified. In some studies, an additional experiment was performed to measure the heat transfer coefficient between the specimen and the fixture [15,27]. When the portion model is taken, the temperature on the virtual boundary replaces the actual contact heat transfer as the thermal boundary condition, which eliminates the need to measure the contact heat transfer coefficient. All the information necessary to construct the thermal model can be obtained by analyzing the thermal image. The portion model can be completed through a single test. This allows the model to be constructed in a more convenient way.

Moreover, it is necessary to identify the conduction loss at the jaws in situ because variable fastening conditions of the specimen will result in a change in the thermal resistance at the jaws [27]. Thanks to the infrared thermal camera, the entire temperature distribution on the specimen surface can be monitored in real time. Naturally, changes in temperature at the two virtual boundaries can also be detected in time. During the fatigue test, the temperature at the virtual boundary indirectly reflects the change of the heat exchange conditions at the end of the specimen. The portion model takes the temperature at two virtual boundaries as the thermal boundary condition. Therefore, the heat exchange between the specimen and the fixture can be reflected in real time by using the portion model proposed in this paper.

### 4.5. Discussion about the Proposed Temperature Data Processing Method 

According to the principle of heat transfer, the temperature of an object depends on the initial conditions, boundary conditions and internal heat source [31]. The initial condition represents the initial temperature distribution of the object. For a real fatigue test, its initial condition is the surface temperature distribution of the specimen at the moment when the cyclic loading starts. The boundary conditions represent the heat exchange between the object and the outside. When the temperature process of the specimen is described by the portion model, the temperature on the two virtual boundaries is the boundary condition. The heat source represents the heat generated inside the object during the heat transfer process. The heat source in the specimen is the intrinsic dissipation caused by the cyclic load.

In this study, the heat source after processing remains the same as that before processing. It means that the processed temperature data retains the same self-heating caused by fatigue as in the real experiment. All fatigue specimens are made of AZ31B magnesium alloy and have the same geometric dimensions. It means that the temperature changes of all specimens under different loads can be described by the same temperature model. After processing by the proposed algorithm, all the temperature data have the same initial conditions ${\overset{=}{\theta }}_{d}\left(x,t=0\right)=0$ and homogeneous thermal boundary conditions ${\overset{=}{\theta }}_{d}\left(x=\pm \frac{L}{2},t\right)=0$, as shown in Equation (7). The heat source is the only variable for the temperature data after processing.

For the fatigue limit evaluation based on self-heating, only the internal heat generation of the material is related to fatigue damage [1,8]. The temperature of the specimen after processing only depends on the self-heating of the experiment material. This makes it a reliable way to indirectly characterize the self-heating caused by fatigue based on the temperature change of the specimen. Judging from the test results, the temperature data after processing can be used to evaluate the fatigue limit of the AZ31B magnesium alloy in both extrusion and transverse directions. This at least shows that the processed temperature data have the ability to reflect the difference between the two self-heating mechanisms of AZ31B under different load levels.

In our previous work, an insulation device applied between the specimen and the fixture was used to control contact heat transfer in an experimental way [26]. Moreover, some researchers installed a circulating cooling device on the fixture of the fatigue testing machine in order to suppress the temperature rise at both ends of the specimen during the test [28]. An ideal experimental condition for temperature-based fatigue studies is expected to be that the temperature at both ends of the specimen does not increase when the cyclic load causes the heating inside the material. In a sense, it is convenient to use the proposed data processing to keep the temperature variation on the boundary of the specimen at zero, because there is no need to install additional temperature control devices.

It should be noted that the temperature data after processing depends on the geometric size of the specimen and the selection of the virtual boundary. The results of specimens of different shapes are not comparable. Only when the temperature of the specimen can be described by the same portion model, the processed temperature data can be used for the fatigue limit assessment. Using heat source intensity is a direct way of measuring self-heating [32]. However, extracting heat source information from the temperature field requires a complicated computer program [32,34].

## 5. Conclusions

In this work, infrared thermographic technology was used to investigate the fatigue process of AZ31B magnesium alloy. According to the experimental results, the following conclusions can be made.

1. By taking the temperature on two virtual boundaries directly as the thermal boundary condition, a special thermal model is established to describe the temperature evolution of the middle portion of specimen during fatigue. This portion model can be completed in one single experiment based on the thermal image information. No more additional tests are required to determine the heat transfer coefficient between the specimen and the fixture.
2. An algorithm is proposed to process the raw temperature data based on the above portion model. This data processing algorithm is based on the mathematical superposition principle. The temperature data after processing are equivalent to the results obtained under the ideal condition where there is no temperature rising at both ends of the specimen. It makes the temperature increment after processing a more reliable representative of the self-heating caused by fatigue.
3. The temperature evolution curve after processing in both extrusion and transverse direction can be divided into three stages before the appearance of macro cracks: the temperature rise stage, the temperature drop stage, and the stable stage. This indicates that the AZ31B magnesium alloy has undergone cyclic hardening in both directions during fatigue.
4. Three different temperature indicators were selected to evaluate the fatigue limit of the AZ31B magnesium alloy based on the evolution curve after processing, namely, the maximum temperature rise, the initial temperature rise slope and the temperature rise of stable stage. From the experimental results, the temperature data processed by the proposed method can be used to estimate the fatigue limit of the AZ31B magnesium alloy. However, the stable temperature does not seem to be a very ideal choice for the AZ31B magnesium alloy.

## Figures and Tables

**Table 1:** Chemical composition of the AZ31B magnesium alloy (wt.%).

Mg	Al	Zn	Mn	Si	Ca	Cu	Fe	Ni
Bal.	2.8	0.7	0.4	0.1	0.04	0.01	0.005	0.001

**Table 2:** Summarized results of fatigue limit obtained by using the Luong method and the Risitano method.

No.	Material	*S*–*N* Curve (MPa)	Indicator	Luong Method		Risitano Method	
				(MPa)	(%)	(MPa)	(%)
1	AZ31B, ED	115	${\overset{=}{\theta }}_{\max}$	109.07	−5.16	104.63	−9.02
2	AZ31B, ED	115	$R_{\theta }$	106.68	−7.23	101.23	−11.97
3	AZ31B, ED	115	${\overset{=}{\theta }}_{\mathrm{stable}}$	133.16	15.79	124.82	8.54
4	AZ31B, TD	105	${\overset{=}{\theta }}_{\max}$	109.40	4.19	106.71	1.63
5	AZ31B, TD	105	$R_{\theta }$	122.21	16.39	118.14	12.51
6	AZ31B, TD	105	${\overset{=}{\theta }}_{\mathrm{stable}}$	64.40	−38.67	48.54	−53.77
